# Regulatory T cell dysfunction in type 1 diabetes: what’s broken and how can we fix it?

**DOI:** 10.1007/s00125-017-4377-1

**Published:** 2017-08-02

**Authors:** Caroline M. Hull, Mark Peakman, Timothy I. M. Tree

**Affiliations:** 1 Programme of Infection and Immunity, Department of Immunobiology, Faculty of Life Sciences and Medicine, King’s College London, Borough Wing, Guy’s Hospital, London, SE1 9RT UK; 2 NIHR Biomedical Research Centre, Guy’s and St Thomas’ NHS Foundation Trust and King’s College London, London, UK

**Keywords:** Immune regulation, Immunotherapy, Review, Tregs, Type 1 diabetes

## Abstract

Type 1 diabetes is an autoimmune disease characterised by the destruction of insulin producing beta cells in the pancreas. Whilst it remains unclear what the original triggering factors for this destruction are, observations from the natural history of human type 1 diabetes, including incidence rates in twins, suggest that the disease results from a combination of genetic and environmental factors. Whilst many different immune cells have been implicated, including members of the innate and adaptive immune systems, a view has emerged over the past 10 years that beta cell damage is mediated by the combined actions of CD4^+^ and CD8^+^ T cells with specificity for islet autoantigens. In health, these potentially pathogenic T cells are held in check by multiple regulatory mechanisms, known collectively as ‘immunological tolerance’. This raises the question as to whether type 1 diabetes develops, at least in part, as a result of a defect in one or more of these control mechanisms. Immunological tolerance includes both central mechanisms (purging of the T cell repertoire of high-affinity autoreactive T cells in the thymus) and peripheral mechanisms, a major component of which is the action of a specialised subpopulation of T cells, known as regulatory T cells (Tregs). In this review, we highlight the evidence suggesting that a reduction in the functional capacity of different Treg populations contributes to disease development in type 1 diabetes. We also address current controversies regarding the putative causes of this defect and discuss strategies to correct it as a means to reduce or prevent islet destruction in a clinical setting.

**Electronic supplementary material:**

The online version of this article (doi:10.1007/s00125-017-4377-1) contains a slide of the figure for download, which is available to authorised users.

## Regulatory T cells: gatekeepers of immunological tolerance

Over the past 20 years it has been established that specific populations of T cells exist and that their primary function is the suppression or regulation of the immune response [1]. Given the generic term ‘regulatory T cells’ (Tregs), these cells form a key part of peripheral immune regulation. A lack of Treg-mediated control has been shown to play a role in numerous autoimmune disorders [2] and in tumour immunology Tregs have been implicated as a mechanism by which tumours evade immune recognition [3].

Regulatory function has been ascribed to a wide variety of different T cell subpopulations, with sometimes confusing nomenclature. In this review, we will adopt recent recommendations and discuss two different populations of CD4^+^ Tregs, delineated based on constitutive expression of the transcription factor forkhead box P3 (FOXP3). More details on Treg nomenclature, generation and function are shown in the Text box.

## CD4^+^FOXP3^+^ Tregs

Perhaps the clearest evidence for a vital role in preventing autoimmunity has been found for a population of CD4^+^ T cells defined by constitutive expression of high levels of CD25 (the IL-2 receptor α chain) and expression of the transcription factor FOXP3. FOXP3^+^ Tregs can either be generated in the thymus (tTregs, previously known as naturally occurring Tregs [nTregs]) or periphery (pTregs, previously called adaptive Tregs [aTregs]) [1, 4]. However, because there are currently no definitive phenotypic markers that can be used to differentiate between these cell types in humans, we will use here the generic term ‘FOXP3^+^ Treg’ to refer to both tTreg and pTreg subtypes. Generation of FOXP3^+^ Tregs depends on the encounter with antigen and signalling via IL-2 [4], a cytokine vital not only for the generation of these cells but also for their survival, expansion and function in the periphery [5, 6]. FOXP3^+^ Tregs exert their suppressive capabilities via several cell-to-cell-contact-dependent and -contact-independent mechanisms. Due to high levels of CD25 expression, FOXP3^+^ Tregs can act as an ‘IL-2 sink’, depriving pathogenic T cells of this growth factor [7]. Suppression can also occur by secretion of suppressive soluble factors, such as TGF-β, IL-10, IL-35 and adenosine, as well as expression of molecules such as lymphocyte-activation gene 3 (LAG-3), cytotoxic T lymphocyte-associated antigen 4 (CTLA-4) and granzyme B [8]. The clearest link between FOXP3^+^ Tregs and autoimmunity comes from the disorder immunodysregulation polyendocrinopathy enteropathy X-linked syndrome (IPEX), in which there are loss-of-function mutations in the *FOXP3* gene [9]. Affected individuals develop a wide range of immunopathology and autoimmune disorders, including type 1 diabetes in >80% of individuals before the age of 2 years. This demonstrates that, if profound, defects in FOXP3^+^ Tregs can elicit type 1 diabetes in most individuals regardless of other genetic or environmental influences, thus pointing to a key role for these cells in maintaining islet-specific tolerance. Similarly, scurfy mice, lacking a functional *Foxp3* gene, display a profoundly dysregulated immune system, including severe generalised autoimmunity, and die of uncontrolled lymphoproliferative disease [10]. Conversely, therapies that increase the number or functional capacity of FOXP3^+^ Tregs can lead to prevention or cure of disease in preclinical models of autoimmunity, including type 1 diabetes [11].
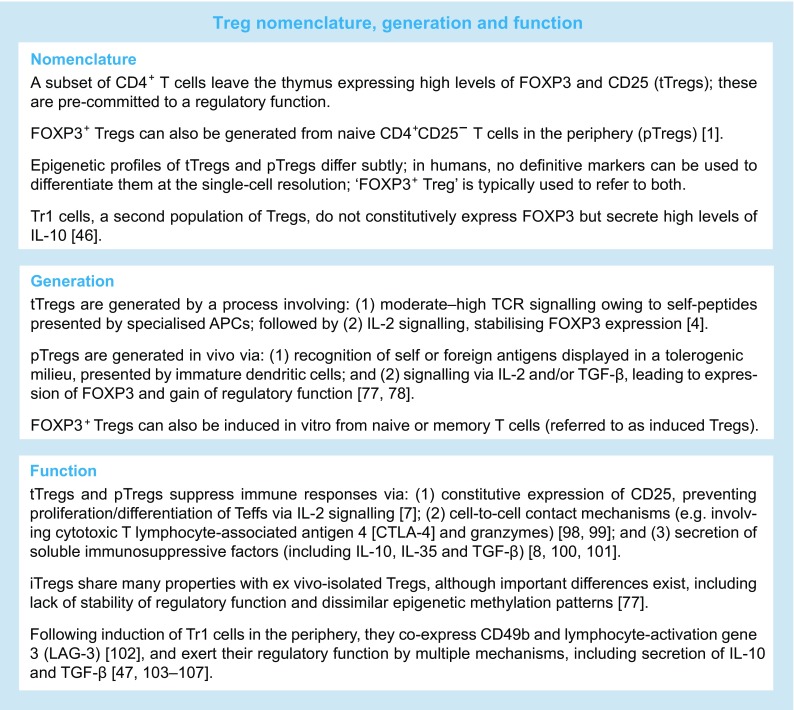


## Defective FOXP3^+^ Treg function: a key immunophenotype in type 1 diabetes

The importance of understanding whether type 1 diabetes is caused by defective immune regulation is clear: not only could it clarify aspects of type 1 diabetes pathogenesis but it could also identify and lead to the development of novel therapeutic interventions or adoptive transfer strategies that specifically strengthen regulatory pathways and, thereby, delay or prevent disease onset in at-risk individuals. Although the defects are not as profound as those seen in individuals affected by IPEX, there is mounting evidence that individuals with polygenic type 1 diabetes display alterations in the fitness and function of FOXP3^+^ Tregs. The theory that such alteration may contribute to disease pathogenesis is supported by the observation that many of the type 1 diabetes susceptibility loci identified by genome-wide association studies may well influence Treg function (e.g. *IL2RA*, *IL2*, *PTPN2*, *CTLA4* and *IL10*) [12], a theme that is discussed in more detail below.

One early report suggested that FOXP3^+^ Tregs (defined as CD4^+^CD25^+^ T cells) were decreased in frequency in individuals with type 1 diabetes (vs control individuals without diabetes) [13]. However, the use of more accurate markers to define these Tregs, including low expression of CD127 and expression of FOXP3, has led to a consensus that the overall frequency of FOXP3^+^ Tregs is unaltered in individuals with type 1 diabetes [14–17]. It is worth noting that these markers are not perfect and that in humans, for example, FOXP3 is transiently upregulated on recently activated effector T cells (Teff), meaning that cells identified by this phenotype are likely to contain a mixture of Tregs and non-regulatory cells [18]. More recently, the selective demethylation of certain regions of the FOXP3 locus (the Treg-specific demethylated region [TSDR]) has been used to identify stable, functionally competent Tregs, allowing their discrimination from activated CD4^+^CD25^+^FOXP3^+^ Teffs [19–21]. However, to date, no difference in the frequency of FOXP3^+^ Tregs has been reported using this or any other enumeration method. Recently, it has also become clear that FOXP3^+^ Tregs are not simply a population of cells sharing a common phenotype but are in fact a heterogeneous mixture of cellular phenotypic subtypes that reflect different states of maturation, differentiation and activation, or use different methods or targets of suppression [22, 23]. It is therefore possible that a shift in the balance or alteration in the frequency of a subtype of Tregs might be present in type 1 diabetes. Indeed Okubo et al recently demonstrated that the frequency of activated FOXP3^+^ Tregs was reduced in individuals with type 1 diabetes when compared with control individuals without diabetes [24].

In contrast to studies examining the frequency of Tregs, there is now a large body of evidence to suggest that FOXP3^+^ Treg function is altered in those with type 1 diabetes. In 2005, Lindley and colleagues reported for the first time that Tregs from individuals with type 1 diabetes were less able to control the proliferation of autologous Teffs than Tregs from HLA- and age-matched control individuals [14], a finding since confirmed by many other researchers [15, 25–28]. Furthermore, not only was suppression of proliferation altered in these co-cultures, but also the balance of cytokines produced was seen to differ: cells from individuals with diabetes produced predominantly proinflammatory cytokines, whereas the co-cultures from individuals without diabetes were dominated by anti-inflammatory cytokines, such as IL-10. Importantly, this reduced suppression is not only present close to diagnosis but is also present in individuals who have had type 1 diabetes for over 20 years. Reduced suppression thus appears to be consistent in type 1 diabetes over time, suggesting that the functional defect represents a stable phenotype. While decreased FOXP3^+^ Treg suppression has been observed independently by several groups, important questions remain regarding the cause, timing and relevance of these findings.

### What causes reduced FOXP3^+^ Treg-mediated suppression?

The reduction in suppression observed in the studies described above could result from changes in either responder or regulatory T cells that were present in the co-cultures. This is a key issue, since many immunotherapy trials are aimed at improving Treg function in those with type 1 diabetes and understanding the nature of the defect is critical for correcting it. This important question has been examined in case–control studies using crossover co-cultures, mixing Tregs and Teffs. These studies observed both effector cell resistance to regulation and reduced Treg suppressive function in type 1 diabetes, with the relative contribution of each phenotype to reduced regulation varying between individuals [27, 28].

In contrast, data from the NOD mouse model of autoimmune diabetes suggest that increasing resistance to Teff regulation with disease progression [29] is the primary cause for reduced suppression. However, there are key differences between type 1 diabetes and the preclinical model. For example, although it has been suggested that a relative deficiency in the strength of IL-2 signalling received by FOXP3^+^ Tregs in both mice and humans may play a key role in their functional deficiency (as discussed below in more detail), in mice this may be mainly driven by polymorphisms in *IL2*, resulting in reduced IL-2 production by Teffs [30], while in humans, in addition to the type 1 diabetes-associated polymorphisms in *IL2*, other disease-associated polymorphisms that confer higher risk are also present in key elements of the IL-2 receptor (*IL2RA* and *IL2RB*) and molecules/phosphatases modulating downstream signalling of IL-2 (e.g. *PTPN2*) [12, 31, 32]. In humans, therefore, the genes that are most relevant to Teff regulation exert their greatest effect in cells reliant upon IL-2 signalling for function and survival, such as Tregs. Thus, in human type 1 diabetes, ‘resistance to regulation’ may also be explained by the inability of Teffs to provide an environment conducive to Treg fitness and function, further compounded by intrinsic Treg defects.

In support of these concepts, in individuals with type 1 diabetes a wide variety of intrinsic differences within the Treg population has been reported, most of which could be viewed as representing less-fit or less-stable FOXP3^+^ Tregs (see Table 1 for details). Alterations in the Treg population in type 1 diabetes include increased levels of Treg apoptosis [25, 26], a decrease in the stability of FOXP3 expression [33, 34] and an increase in the frequency of Tregs that produce proinflammatory cytokines, such as IFN-γ and IL-17 [35, 36]. More recently, in an elegant study, Pesenacker and colleagues examined the expression of a panel of FOXP3^+^ Treg-specific transcripts in Tregs freshly isolated from individuals with recent-onset type 1 diabetes and well-matched individuals without diabetes [37]. They identified a panel of six genes, including *FOXP3*, *TNFRSF1B* (*CD120b*) and *LRRC32* (*GARP*), which were directly linked to Treg function and stability and were differentially expressed in Tregs from individuals with diabetes. Similarly, other studies have identified subtle differences in gene expression profiles in Tregs according to type 1 diabetes presence or absence [38].

**Table 1:** Intrinsic differences within the Treg population in type 1 diabetes

Treg immunophenotype observed	Study authors (date)	Individuals studied	Study outcomes
Reduced Treg IL-2 sensitivity	Long et al (2011) [32]	NDB, stratified by *PTPN22* genotype	The T1D-associated genotype was associated with reduced IL-2 signalling
	Garg et al (2012) [34]	NDB stratified by *IL2RA* genotype	The T1D-associated genotype was associated with reduced IL-2 signalling
	Yang et al (2015) [39]	With long-standing T1D	Reduced IL-2 signalling was associated with the T1D-associated *PTPN2* genotype and lower levels of Treg-mediated suppression
	Cerosaletti et al (2013) [95]	With T1D; NDB but at risk	Reduced IL-2 signalling was observed in T1D vs NDB; IL-2 signalling was reduced in NDB with T1D-associated *PTPN2* and *IL2RA* genotypes
	Long et al (2010) [33]	With T1D; NDB	Reduced IL-2 signalling was observed in T1D vs NDB
Unstable FOXP3 expression	Long et al (2010) [33]	With T1D; NDB	Reduced FOXP3 expression under conditions of limiting IL-2 in individuals with T1D vs NDB
	Garg et al (2012) [34]	NDB stratified by *IL2RA* genotype	The T1D-associated genotype was associated with reduced FOXP3 expression under conditions of limiting IL-2
Increased Treg apoptosis	Glisic-Milosavljevic et al (2007) [26]	With recent-onset and long-standing T1D; islet AAb^+^ (at-risk); NDB	Increased Treg apoptosis was observed in recent-onset T1D and at-risk individuals with two or three AAbs when compared to low risk individuals and NDB
	Glisic-Milosavljevic et al (2007) [25]	With new-onset T1D; NDB	Longitudinal study showing increased levels of Treg apoptosis close to diagnosis of T1D vs NDB, but this diminished over time
	Glisic et al (2009) [41, 96]	With recent-onset T1D; with long-standing T1D; NDB	Increased levels of Treg apoptosis was observed in recent-onset T1D vs NDB and associated with the high-risk *HLA*-*DQB1* haplotype
Increased Treg proinflammatory cytokine secretion	McClymont et al (2011) [35]	With established T1D; NDB	Increased frequency of IFN-γ-producing Tregs in individuals with T1D vs NDB; these Tregs displayed reduced suppressive function compared with non-IFN-γ-producing Tregs
	Marwaha et al (2010) [36]	With recent-onset T1D; NDB	Increased frequency of IL-17-producing cells in CD45RA^−^CD25^int^FOXP3^low^ T cells vs NDB, which displayed reduced suppressive function
Altered Treg transcriptome	Pesenacker et al (2016) [37]	With recent-onset T1D; with established T1D; NDB	Identified a panel of genes that are differentially expressed in Tregs from children with recent-onset T1D vs NDB
	Ferraro et al (2014) [38]	With established T1D, with T2D; NDB	A number of genes were shown to have reduced expression in individuals with T1D vs those without

AAb, autoantibody; NDB, not diabetic; T1D, type 1 diabetes; T2D, type 2 diabetes

Given the key role that IL-2 signalling plays in maintaining FOXP3 expression, thereby maintaining Treg fitness, it has been postulated that many of the Treg-intrinsic defects observed in type 1 diabetes may be caused by a relative reduction in IL-2 signalling. Indeed, transcriptional profiles of Tregs from individuals with recent-onset diabetes share many features with IL-2-starved, apoptosis-prone Tregs. Yang and colleagues recently linked many of these associations together for the first time, demonstrating that individuals with type 1 diabetes and low IL-2 signalling had Tregs that were less able to maintain FOXP3 expression under limiting concentrations of IL-2 and displayed reduced suppressor function [39]. Although our knowledge of factors that influence FOXP3^+^ Treg stability and function has increased rapidly over the past few years, and the possibility that differential IL-2 signalling may explain at least some of the differences seen in those with type 1 diabetes, a full understanding of the precise molecular basis underlying FOXP3^+^ Treg dysfunction in type 1 diabetes is still lacking and warrants further investigation. In summary, the fitness and function of Tregs and Teffs may be inextricably linked. However, phenotypic differences are clearly observable in Tregs when comparing those from individuals with and without type 1 diabetes, irrespective of whether this is primarily a case of ‘nature’ or ‘nurture’.

### Is reduced FOXP3^+^ Treg function a cause or effect of disease?

To better understand how tolerance is lost in type 1 diabetes, a key issue to address is whether the decreased suppressive capability of Tregs is due to changes in the immune system that are caused by development of type 1 diabetes or whether Treg dysfunction is involved in disease initiation. Studies examining Treg function in individuals with stage 1 diabetes, as defined by autoantibody positivity, suggest that Treg defects pre-date clinical disease, supporting a causative role for Treg dysfunction [26, 40]. However, interpretation of these results is not straightforward because although these individuals do not show overt diabetes, they may already have islet inflammation which could influence Treg function. An alternative approach is to assess Treg fitness and function in individuals who possess a high-risk haplotype for type 1 diabetes but who have no evidence of disease. These genotype–phenotype studies rest on the hypothesis that if a type 1 diabetes susceptibility genotype is associated with altered Treg function, then Treg dysfunction is likely to be causal in type 1 diabetes. To date, such studies have demonstrated that polymorphisms in *IL2RA* and *PTPN2* are indeed associated with reduced Treg fitness and/or function in the absence of disease [32, 34]. These observations in individuals without diabetes are supported by similar genotype–phenotype studies in individuals with type 1 diabetes, including the associations between Treg IL-2 sensitivity and *IL2RA* genotype [39] and between Treg apoptosis and HLA class II haplotype [41]. While these studies all support a causative role for Treg dysfunction in type 1 diabetes, to fully understand the timing of Treg dysfunction and its relationship with disease progression, longitudinal studies are required that follow individuals at high risk through the stages of type 1 diabetes. Such studies may lead to the correlation of Treg function with the breakdown of immunological tolerance, the emergence of activated autoreactive T cells and the progression to beta cell destruction.

### Where does the imbalance in FOXP3^+^ Treg function occur?

The studies discussed so far demonstrate reduced FOXP3^+^ Treg function in type 1 diabetes, but an important caveat is that their conclusions are drawn based on a phenotype found in circulating peripheral Tregs rather than Tregs present at the site of tissue damage. Studies in the NOD mouse have highlighted the fact that Treg dysfunction is mainly limited to the pancreas and draining lymph nodes. In this model of type 1 diabetes, as the disease develops the frequency of Tregs increases in the pancreatic draining lymph node (PLN) but decreases in the pancreas, with reduced Treg CD25 expression and an increase in apoptosis being observed. Successful treatment of NOD mice by IL-2 therapy, leading to reversal of disease, specifically prevents the loss of Tregs in the pancreas [11], demonstrating the importance of studying Tregs from the site of tissue damage.

Although such studies are not easily performed in human type 1 diabetes, relevant observations have been made using tissue recovered from donor cadavers. Interestingly, these studies have revealed important differences between mouse and human insulitis. Most notably, infiltration in human islets is far less florid than seen in mouse islets and rarely contains any FOXP3^+^ Tregs, suggesting that regulation of the immune response takes place at another location [42]. In this regard, an important study by Ferraro and colleagues revealed differences in Tregs from the PLN of individuals with type 1 diabetes [43]. These investigators observed decreased levels of suppression by Tregs obtained from the PLN of individuals with type 1 diabetes and an increase in secretion of the proinflammatory cytokine IL-17. These studies demonstrate the importance of tissue-specific investigations and suggest that the PLN may be a key site of Treg dysfunction in type 1 diabetes. Furthermore, detailed studies using cells isolated from a variety of anatomical sites, such as those available via the JDRF-sponsored Network for Pancreatic Organ donors with Diabetes programme (nPOD), will be vital for gaining a deeper insight into immune dysregulation in type 1 diabetes closer to the target organ.

### Is reduced FOXP3^+^ Treg function universal in type 1 diabetes?

Another topic worthy of discussion is the degree of heterogeneity of Treg function observed in all studies to date. From the studies described above, one could conclude that reduced Treg function plays a role in all type 1 diabetes development. However, it is worth noting that, to date, all studies examining FOXP3^+^ Treg function have found a large degree of overlap between individuals with and without type 1 diabetes, with only a subgroup of individuals with type 1 diabetes clearly displaying the immune phenotype associated with poor function. This suggests that the reduced Treg function observed using these assays may be restricted to, or more easily revealed in, a subset of individuals with type 1 diabetes. Understanding how to stratify individuals in terms of the specific defects that have led to an imbalanced immune response will be critical when deciding who is likely to benefit from a given immunotherapy. An example highlighting the heterogeneity seen within type 1 diabetes cohorts demonstrated variation in the IL-2 sensitivity of Tregs from different individuals [44]. Those with reduced Treg IL-2 sensitivity had unstable FOXP3 expression and poor suppressor capabilities and it is possible that these individuals would benefit from IL-2 immunotherapy. As we continue to develop our understanding of the heterogeneity present within individuals with type 1 diabetes, it is important to test potential therapies in those who are most likely to benefit from a given treatment, highlighting the need for a personalised approach to immunotherapy in type 1 diabetes.

## CD4^+^FOXP3^−^ Treg cells

In addition to FOXP3^+^ Tregs, other subsets of CD4 T cells with regulatory properties have been described, including one characterised by secretion of high levels of IL-10 upon recognition of cognate peptide. Often referred to as T regulatory type 1 (Tr1) Tregs, these cells were first described in individuals who developed tolerance following HLA-mismatched haematopoietic stem-cell transplantation [45]. Tr1 cells are capable of suppressing T cell responses and modulating antigen-presenting cell (APC) function via a variety of mechanisms, including expression of inhibitory cell surface receptors, cytolytic activity and secretion of soluble factors [46]. The mechanism by which Tr1 cells are naturally induced in vivo remains poorly understood, although evidence from mouse models and human studies suggests that they are generated from naive CD4 T cells upon repeated stimulation with self or foreign antigens presented by immature or tolerogenic dendritic cells. Numerous experimental models have demonstrated that Tr1 cells play a key role in maintaining tolerance to both self-antigens and gut microbiota [46]. Furthermore, defects in the number and/or functional potential of Tr1 (or Tr1-like) cells have been implicated in the pathogenesis of a range of human autoimmune [47–49] and allergic conditions [50].

### Islet-specific IL-10-secreting cells in type 1 diabetes

A mounting body of evidence now suggests that islet-specific Tr1-like cells may play an important role in the development of type 1 diabetes. In 2004 Arif and colleagues identified a novel population of naturally arising CD4^+^ T cells that secrete IL-10 following exposure to islet autoantigens [51]. Subsequent isolation and functional characterisation of these naturally occurring islet-specific T cells from individuals without diabetes demonstrated that they share many properties with Tr1 cells and exert a potent regulatory function. In vitro, this regulatory function is primarily mediated by the specific destruction of APCs presenting islet peptides. This mechanism prevents activation of proinflammatory T cells by the same APC and, if operational in vivo, would represent a potentially important mechanism of maintaining antigen-specific tolerance. Studies investigating the frequency of these cells in individuals with varying backgrounds of islet autoimmunity have made several important observations. First, these cells are enriched in those at risk of type 1 diabetes but with no evidence of pathogenic islet autoimmunity, such as individuals without diabetes but carrying high-risk HLA class II molecules [51]. Second, IL-10-secreting Tregs that are observed in those with type 1 diabetes are associated with less-aggressive autoimmunity as demonstrated by a reduced magnitude of proinflammatory islet-specific T cells and fewer autoantibodies [52], a later age of onset [51] and superior glycaemic control after diagnosis [53]. Third, although the overall frequency of islet-specific IL-10-secreting T cells does not differ between those with type 1 diabetes and autoantibody-negative first-degree relatives (FDRs), cells from FDRs were observed to secrete more IL-10, suggesting potential functional differences in these cells; this warrants further investigation [54]. Taken together, these data suggest that islet-specific IL-10-secreting cells are associated with protection from pathological islet autoimmunity and offer a potentially powerful method by which to strengthen tolerance in an antigen-specific manner.

## Promoting immune regulation in type 1 diabetes

**Fig. 1:**
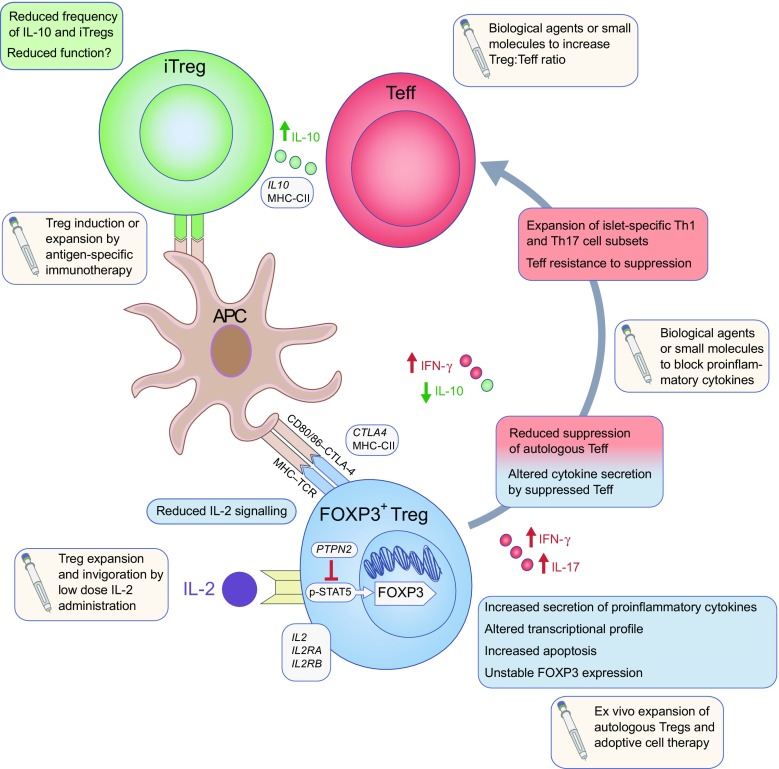
Alterations in Treg phenotype and function observed in type 1 diabetes. FOXP3^+^ Tregs from individuals with type 1 diabetes are less able to control the proliferation of and cytokine production by effector CD4^+^ T cells compared with those from individuals without diabetes. This defective regulation may be owing to two non-mutually exclusive factors: differences in the Teff population (shown in red boxes) and/or Treg intrinsic defects (shown in blue boxes) (where differences overlap, details are shown in red/blue boxes). Additionally, the frequency and function of induced Tregs (iTregs) may play a role in promoting imbalance of the immune system in type 1 diabetes (green boxes). In many cases, these immunophenotypes may be influenced by gene polymorphisms associated with type 1 diabetes susceptibility (shown in grey boxes). Potential avenues for strengthening immune regulation by Treg invigoration are indicated in beige boxes. Red circles, IFN-γ/IL-17; green circles, IL-10. The grey arrow represents how unstable expression profiles of FOXP3 by Tregs increases the production of proinflammatory cytokines, promoting the function and expansion of islet-destructive Teff cells. MHC-CII, *HLA-DRB1*/*HLA-DQA1*/*HLA-DQB1*; STAT, signal transducer and activator of transcription; Th, T helper

Despite heterogeneity within type 1 diabetes cohorts, promoting immune regulation, even in individuals who do not have reduced Treg frequency or function, may tip the balance of the immune response enough to promote protection of beta cells. Evidence is mounting from clinical studies in type 1 diabetes and other conditions characterised by immune dysregulation that such therapeutic approaches might have an impact upon established and developing disease (see Fig. 1 for a summary of Treg defects and current immunotherapies aimed at strengthening immune regulation).

### Agents that alter the balance of effector:regulatory T cells

Several monoclonal antibodies and small-molecule therapies that were initially developed to treat other diseases have been found to demonstrate clinical benefit in type 1 diabetes and may operate by altering Treg frequency or function. Treatment of individuals with type 1 diabetes with alefacept (a lymphocyte function-associated antigen-3 immunoglobulin [LFA-3Ig] fusion protein that binds to CD2 and depletes T cells displaying high levels of this surface antigen) significantly decreased dependency on exogenous insulin 24 months after treatment [55]. This effect correlated with an increase in the ratio of FOXP3^+^ Tregs to CD4^+^ and CD8^+^ effector and central memory T cells. In another study, combination therapy with antithymocyte globulin and granulocyte colony stimulating factor increased or preserved beta cell function in individuals with type 1 diabetes when measured 1 year following treatment [56] and this was associated with a higher frequency of FOXP3^+^ Tregs. Taken together, these studies support further investigation of the therapeutic potential of Tregs in type 1 diabetes.

Monoclonal antibody therapies blocking proinflammatory cytokines may also represent a method by which Treg function can be promoted. It is well known that Treg-mediated suppression can be reduced in the presence of pro-inflammatory cytokines, such as IL-6. The potential of anti-IL-6 therapy has previously been demonstrated in a variety of conditions, including systemic lupus erythematosus and Crohn’s disease (reviewed by Nepom et al [57]) and a clinical trial testing anti-IL6 therapy in type 1 diabetes has begun (ClinicalTrials.gov registration no. NCT02293837) [58].

### Direct targeting of FOXP3^+^ Tregs by IL-2 administration

Clinical trials have now begun testing therapies that are specifically designed to promote the expansion or function of FOXP3^+^ Tregs in type 1 diabetes. One such strategy, supported by observations on Treg dysfunction, is to use exogenously administered low-dose IL-2 to selectively promote Treg function with the rationale that Tregs respond to lower doses of IL-2 compared with other cells of the immune system because of their high expression levels of CD25 [59]. Similar studies in other conditions marked by immune dysregulation, including chronic graft-versus-host disease (GvHD) [60, 61], hepatitis C virus-induced vasculitis [62], systemic lupus erythematosus [63] and alopecia areata [64], have been conducted with encouraging results. A Phase I/II clinical trial in type 1 diabetes [65] demonstrated the safety of low-dose IL-2 administration and an increase in the frequency of pTregs was observed. Similar studies in other conditions marked by immune dysregulation have shown great promise and clinical benefit in some individuals. A second clinical trial, conducted by Diabetes TrialNet and the Immune Tolerance Network, used higher doses of IL-2 in combination with the inhibitor of the mammalian target of rapamycin (mTOR), rapamycin. While this therapy led to an increase in Treg frequency, it also induced a transient reduction in beta cell function, possibly owing to off-target effects on other cell populations such as natural killer (NK) cells [66, 67]. This study clearly highlights the importance of carefully assessing the dose and frequency of IL-2 administration to selectively target Tregs while avoiding unwanted off-target effects. These issues are being investigated intensively in mechanistic studies with immunological endpoints prior to conducting fully powered Phase II efficacy trials [68, 69].

### Adoptive Treg cell therapy

An alternative method to promote immune regulation by Tregs is to increase their frequency by adoptively transferring autologous Treg populations. Recent advances in cell sorting allow for the isolation of highly pure FOXP3^+^ Tregs, under conditions of good manufacturing practice, using a cell surface phenotype of CD4^+^CD25^high^CD127^low^. Subsequent polyclonal stimulation of isolated FOXP3^+^ Tregs ex vivo leads to the expansion of billions of cells from a single blood draw, allowing for their therapeutic potential to be explored. The first clinical trial applying adoptive polyclonal Treg therapy to type 1 diabetes was completed in 2012 [70]. Administration of autologous, expanded CD4^+^CD25^high^CD127^low^ Tregs to children within 2 months of type 1 diabetes diagnosis significantly increased pTreg frequency, coinciding with a decrease in dependency on exogenous insulin. A 1 year follow-up study showed that 8 out of 12 children treated with Tregs required less exogenous insulin and two children were independent of exogenous insulin [71]. A second Phase I safety trial in individuals diagnosed with type 1 diabetes within 2 years of recruitment was also completed in 2015, further demonstrating the safety and feasibility of this approach [72].

While the initial clinical trials using polyclonal Treg therapy in type 1 diabetes demonstrate the feasibility and safety of this approach, studies in the NOD mouse suggest that islet antigen-specific Tregs would be more efficacious as a therapy [73–75]. Large populations of murine antigen-specific Tregs can be produced with ease using T cell receptor (TCR)-transgenic mice, but in humans this is difficult as islet antigen-specific Tregs within the pTreg pool are very rare. Selective expansion of antigen-specific Tregs has been used to produce alloantigen-specific populations to treat GvHD. Tregs specific for alloantigens presented by donor-derived B cells stimulated with CD40 ligand were successfully expanded to clinically relevant numbers [76]. This approach is unlikely to be successful in type 1 diabetes as there are fewer antigens involved in the autoimmune response and, therefore, Tregs with a relevant specificity have an even lower frequency. Expansion of all islet antigen-specific CD4^+^ T cells could also be a strategy, since stimulation of Teffs via their TCR has been found to induce a subpopulation of cells with regulatory potential [77] and the presence of TGF-β in cultures has also been shown to induce Treg populations [78]. Using culture conditions to skew T cells towards a regulatory phenotype has the disadvantage that once cells are adoptively transferred, the stability of their regulatory phenotype is unknown. One option to improve the stability of Treg populations is by ectopic expression of FOXP3 to achieve a homogeneous FOXP3^+^ Treg population with potent regulatory potential [79, 80]. An alternative that has received a great deal of attention in both autoimmunity and cancer therapy is the redirection of T cell specificity using TCR gene therapy. In type 1 diabetes, the antigen specificity of polyclonal Treg populations could be redirected towards islet antigens to produce large populations of islet antigen-specific Tregs. A proof of principle study has indeed demonstrated that human Treg antigen specificity can be redirected by TCR gene transfer [81].

Further development of adoptive Treg therapy may need to consider the homing potential of Treg populations in addition to their antigen specificities. It has previously been demonstrated in the NOD mouse that adoptive transfer of CD62L^+^ but not CD62L^−^ Tregs inhibited type 1 diabetes development. In humans, isolation of CD45RA^+^ rather than CD45RA^−^ Tregs produced a homogeneous population of Tregs that expressed lymph node homing receptors, including CCR7 and CD62L [22, 82]. The use of drugs, such as rapamycin and all-trans retinoic acid (ATRA), influences the homing signatures of human Treg populations produced for adoptive cell therapy. Tregs expanded in the presence of rapamycin express skin homing receptors, such as CCR4, while those expanded in the presence of ATRA express gut homing receptors, such as α4β7 integrin [83]. Expansion using a combination of both drugs produces a Treg population with a diverse range of homing receptors. Together, these data provide an insight into how isolation and expansion methods can be used to ‘imprint’ different homing profiles on Treg populations, adding an additional level of control. The use of adoptive Treg therapy in type 1 diabetes may be in its infancy but recent advances in the fields of cancer and transplantation demonstrate that adoptive cell therapies may hold great promise for rebalancing the human immune system.

### Expanding islet-specific Tregs by antigen-specific immunotherapy

It has long been acknowledged that administration of antigens or peptides under tolerogenic conditions has the potential to induce or expand populations of antigen-specific Tregs capable of modulating disease. In animal models of type 1 diabetes, administration of islet autoantigen using a variety of tolerogenic regimens has provided protection against islet destruction, which is often associated with an increase in IL-10 production by CD4^+^ T cells, although in many cases the regulatory potential of these cells is not well understood [84–86]. More recently, in a humanised HLA-transgenic mouse model of islet autoimmunity, Gibson and colleagues demonstrated that, while peptide presented by tolerogenic dendritic cells controlled autoimmunity and was associated with islet-specific IL-10 production, intradermal injection of the same peptide also reduced autoimmunity and increased the proliferation of FOXP3^+^ Tregs [87]. This elegantly demonstrates that the route and method of delivery of an antigen-specific immunotherapy can influence the mechanism by which it may afford protection. In human type 1 diabetes, administration of the islet autoantigen GAD65 in alum resulted in some preservation of islet function in new-onset type 1 diabetes in Phase II trials [88] but failed to meet its primary endpoints in Phase III trials [89]. Treatment was associated with increased expression of FOXP3 in T cells stimulated with GAD65 ex vivo, although this response was not associated with preserved C-peptide and it was unclear whether it reflected an increase in bona fide FOXP3^+^ Tregs or activated Teffs [90]. In a 2009 Phase I study in individuals with type 1 diabetes, those who were given low doses of proinsulin peptide showed an increase in peptide-specific IL-10 responses when compared with individuals given placebo, demonstrating proof of concept [91]. Other trials using islet peptides representing known epitopes recognised by CD4^+^ T cells are ongoing. Several of these involve novel methods of delivery aimed at increasing the potential to induce Treg responses, including loading peptide onto tolerogenic dendritic cells [92] or conjugating the peptides to nanoparticles [93], and appear to induce populations of Tregs with similar properties to the naturally occurring IL-10-secreting cells described above [94, 95].

Compared with the progress in other fields (such as allergy), antigen-specific immunotherapy in type 1 diabetes may still be in its infancy. However, it remains a potentially powerful weapon that has the potential to specifically control islet autoimmunity, thereby avoiding many of the potential adverse events that may be associated with more generalised immunosuppression.

## Conclusions

Partly fuelled by observations of diminished Treg function or frequency in type 1 diabetes, the strengthening of immunoregulation by Treg invigoration is a major area of clinical trial activity. However, despite the focus of several high-profile clinical studies on increasing Tregs via therapeutic intervention, key questions remain unanswered: when and precisely how do changes in Treg populations arise? How can we best identify individuals with dysfunctional Tregs? Who will benefit from particular forms of immunotherapy? What are the best ways to increase Treg frequency or function? Gaining a better understanding of the natural history of Treg function in type 1 diabetes and unravelling the molecular profile of functional and dysfunctional Treg subsets has the potential to increase our understanding of the molecular basis of type 1 diabetes and may reveal new targets for immunotherapy. These studies may also identify biomarkers that can be deployed in ongoing clinical trials and ultimately offer the potential to stratify individuals who may benefit most from Treg-strengthening therapies.

## Electronic supplementary material

ESM Downloadable slide (PPTX 300 kb)

## Abbreviations

APC: Antigen-presenting cell
ATRA: All-trans retinoic acid
FDR: First-degree relative
FOXP3: Forkhead box P3
GvHD: Graft-versus-host disease
IPEX: Immunodysregulation polyendocrinopathy enteropathy X-linked syndrome
PLN: Pancreatic draining lymph node
pTreg: Regulatory T cell generated in the periphery
TCR: T cell receptor
Teff: Effector T cell
Tr1: T regulatory type 1
Treg: Regulatory T cell
tTreg: Regulatory T cell generated in the thymus

## References

[1] Ohkura N, Kitagawa Y, Sakaguchi S (2013). Development and maintenance of regulatory T cells. Immunity.

[2] Grant CR, Liberal R, Mieli-Vergani G, Vergani D, Longhi MS (2015). Regulatory T-cells in autoimmune diseases: challenges, controversies and--yet--unanswered questions. Autoimmun Rev.

[3] Curiel TJ (2007). Tregs and rethinking cancer immunotherapy. J Clin Invest.

[4] Lio CW, Hsieh CS (2008). A two-step process for thymic regulatory T cell development. Immunity.

[5] Malek TR, Bayer AL (2004). Tolerance, not immunity, crucially depends on IL-2. Nat Rev Immunol.

[6] Zorn E, Nelson EA, Mohseni M (2006). IL-2 regulates FOXP3 expression in human CD4+CD25+ regulatory T cells through a STAT-dependent mechanism and induces the expansion of these cells in vivo. Blood.

[7] Busse D, de la Rosa M, Hobiger K (2010). Competing feedback loops shape IL-2 signaling between helper and regulatory T lymphocytes in cellular microenvironments. Proc Natl Acad Sci U S A.

[8] Sojka DK, Huang YH, Fowell DJ (2008). Mechanisms of regulatory T-cell suppression—a diverse arsenal for a moving target. Immunology.

[9] Wildin RS, Ramsdell F, Peake J (2001). X-linked neonatal diabetes mellitus, enteropathy and endocrinopathy syndrome is the human equivalent of mouse scurfy. Nat Genet.

[10] Brunkow ME, Jeffery EW, Hjerrild KA (2001). Disruption of a new forkhead/winged-helix protein, scurfin, results in the fatal lymphoproliferative disorder of the scurfy mouse. Nat Genet.

[11] Grinberg-Bleyer Y, Baeyens A, You S (2010). IL-2 reverses established type 1 diabetes in NOD mice by a local effect on pancreatic regulatory T cells. J Exp Med.

[12] Todd JA, Walker NM, Cooper JD (2007). Robust associations of four new chromosome regions from genome-wide analyses of type 1 diabetes. Nat Genet.

[13] Kukreja A, Cost G, Marker J (2002). Multiple immuno-regulatory defects in type-1 diabetes. J Clin Invest.

[14] Lindley S, Dayan CM, Bishop A, Roep BO, Peakman M, Tree TI (2005). Defective suppressor function in CD4(+)CD25(+) T-cells from patients with type 1 diabetes. Diabetes.

[15] Brusko TM, Wasserfall CH, Clare-Salzler MJ, Schatz DA, Atkinson MA (2005). Functional defects and the influence of age on the frequency of CD4+ CD25+ T-cells in type 1 diabetes. Diabetes.

[16] Brusko T, Wasserfall C, McGrail K (2007). No alterations in the frequency of FOXP3+ regulatory T-cells in type 1 diabetes. Diabetes.

[17] Putnam AL, Vendrame F, Dotta F, Gottlieb PA (2005). CD4+CD25high regulatory T cells in human autoimmune diabetes. J Autoimmun.

[18] Wang J, Ioan-Facsinay A, van der Voort EI, Huizinga TW, Toes RE (2007). Transient expression of FOXP3 in human activated nonregulatory CD4+ T cells. Eur J Immunol.

[19] Polansky JK, Schreiber L, Thelemann C (2010). Methylation matters: binding of Ets-1 to the demethylated Foxp3 gene contributes to the stabilization of Foxp3 expression in regulatory T cells. J Mol Med (Berl).

[20] Kim HP, Leonard WJ (2007). CREB/ATF-dependent T cell receptor-induced FoxP3 gene expression: a role for DNA methylation. J Exp Med.

[21] Floess S, Freyer J, Siewert C (2007). Epigenetic control of the foxp3 locus in regulatory T cells. PLoS Biol.

[22] Miyara M, Yoshioka Y, Kitoh A (2009). Functional delineation and differentiation dynamics of human CD4+ T cells expressing the FoxP3 transcription factor. Immunity.

[23] Mason GM, Lowe K, Melchiotti R (2015). Phenotypic complexity of the human regulatory T cell compartment revealed by mass cytometry. J Immunol.

[24] Okubo Y, Torrey H, Butterworth J, Zheng H, Faustman DL (2016). Treg activation defect in type 1 diabetes: correction with TNFR2 agonism. Clin Transl Immunol.

[25] Glisic-Milosavljevic S, Wang T, Koppen M (2007). Dynamic changes in CD4+ CD25+(high) T cell apoptosis after the diagnosis of type 1 diabetes. Clin Exp Immunol.

[26] Glisic-Milosavljevic S, Waukau J, Jailwala P (2007). At-risk and recent-onset type 1 diabetic subjects have increased apoptosis in the CD4+CD25+ T-cell fraction. PLoS One.

[27] Lawson JM, Tremble J, Dayan C (2008). Increased resistance to CD4+CD25hi regulatory T cell-mediated suppression in patients with type 1 diabetes. Clin Exp Immunol.

[28] Schneider A, Rieck M, Sanda S, Pihoker C, Greenbaum C, Buckner JH (2008). The effector T cells of diabetic subjects are resistant to regulation via CD4+ FOXP3+ regulatory T cells. J Immunol.

[29] D'Alise AM, Auyeung V, Feuerer M (2008). The defect in T-cell regulation in NOD mice is an effect on the T-cell effectors. Proc Natl Acad Sci U S A.

[30] Sgouroudis E, Albanese A, Piccirillo CA (2008). Impact of protective IL-2 allelic variants on CD4+ Foxp3+ regulatory T cell function in situ and resistance to autoimmune diabetes in NOD mice. J Immunol.

[31] Dendrou CA, Wicker LS (2008). The IL-2/CD25 pathway determines susceptibility to T1D in humans and NOD mice. J Clin Immunol.

[32] Long SA, Cerosaletti K, Wan JY (2011). An autoimmune-associated variant in PTPN2 reveals an impairment of IL-2R signaling in CD4(+) T cells. Genes Immun.

[33] Long SA, Cerosaletti K, Bollyky PL (2010). Defects in IL-2R signaling contribute to diminished maintenance of FOXP3 expression in CD4(+)CD25(+) regulatory T-cells of type 1 diabetic subjects. Diabetes.

[34] Garg G, Tyler JR, Yang JH (2012). Type 1 diabetes-associated IL2RA variation lowers IL-2 signaling and contributes to diminished CD4+CD25+ regulatory T cell function. J Immunol.

[35] McClymont SA, Putnam AL, Lee MR (2011). Plasticity of human regulatory T cells in healthy subjects and patients with type 1 diabetes. J Immunol.

[36] Marwaha AK, Crome SQ, Panagiotopoulos C (2010). Cutting edge: increased IL-17-secreting T cells in children with new-onset type 1 diabetes. J Immunol.

[37] Pesenacker AM, Wang AY, Singh A (2016). A regulatory T-cell gene signature is a specific and sensitive biomarker to identify children with new-onset type 1 diabetes. Diabetes.

[38] Ferraro A, D'Alise AM, Raj T (2014). Interindividual variation in human T regulatory cells. Proc Natl Acad Sci U S A.

[39] Yang JH, Cutler AJ, Ferreira RC (2015). Natural variation in interleukin-2 sensitivity influences regulatory T-cell frequency and function in individuals with long-standing type 1 diabetes. Diabetes.

[40] Jana S, Campbell H, Woodliff J (2010). The type of responder T-cell has a significant impact in a human in vitro suppression assay. PLoS One.

[41] Glisic S, Jailwala P (2012). Interaction between Treg apoptosis pathways, Treg function and HLA risk evolves during type 1 diabetes pathogenesis. PLoS One.

[42] Willcox A, Richardson SJ, Bone AJ, Foulis AK, Morgan NG (2009). Analysis of islet inflammation in human type 1 diabetes. Clin Exp Immunol.

[43] Ferraro A, Socci C, Stabilini A (2011). Expansion of Th17 cells and functional defects in T regulatory cells are key features of the pancreatic lymph nodes in patients with type 1 diabetes. Diabetes.

[44] Yang M, Wang X, Huang H (2016). Natural variation of H3K27me3 modification in two Arabidopsis accessions and their hybrid. J Integr Plant Biol.

[45] Roncarolo MG, Yssel H, Touraine JL, Betuel H, De Vries JE, Spits H (1988). Autoreactive T cell clones specific for class I and class II HLA antigens isolated from a human chimera. J Exp Med.

[46] Roncarolo MG, Gregori S, Bacchetta R, Battaglia M (2014). Tr1 cells and the counter-regulation of immunity: natural mechanisms and therapeutic applications. Curr Top Microbiol Immunol.

[47] Veldman C, Hohne A, Dieckmann D, Schuler G, Hertl M (2004). Type I regulatory T cells specific for desmoglein 3 are more frequently detected in healthy individuals than in patients with pemphigus vulgaris. J Immunol.

[48] Ward FJ, Hall AM, Cairns LS (2008). Clonal regulatory T cells specific for a red blood cell autoantigen in human autoimmune hemolytic anemia. Blood.

[49] Gianfrani C, Levings MK, Sartirana C (2006). Gliadin-specific type 1 regulatory T cells from the intestinal mucosa of treated celiac patients inhibit pathogenic T cells. J Immunol.

[50] Meiler F, Zumkehr J, Klunker S, Ruckert B, Akdis CA, Akdis M (2008). In vivo switch to IL-10-secreting T regulatory cells in high dose allergen exposure. J Exp Med.

[51] Arif S, Tree TI, Astill TP (2004). Autoreactive T cell responses show proinflammatory polarization in diabetes but a regulatory phenotype in health. J Clin Invest.

[52] Arif S, Leete P, Nguyen V (2014). Blood and islet phenotypes indicate immunological heterogeneity in type 1 diabetes. Diabetes.

[53] Sanda S, Roep BO, von Herrath M (2008). Islet antigen specific IL-10+ immune responses but not CD4+CD25+FoxP3+ cells at diagnosis predict glycemic control in type 1 diabetes. Clin Immunol.

[54] Petrich de Marquesini LG, Fu J, Connor KJ (2010). IFN-gamma and IL-10 islet-antigen-specific T cell responses in autoantibody-negative first-degree relatives of patients with type 1 diabetes. Diabetologia.

[55] Rigby MR, Harris KM, Pinckney A (2015). Alefacept provides sustained clinical and immunological effects in new-onset type 1 diabetes patients. J Clin Invest.

[56] Haller MJ, Gitelman SE, Gottlieb PA (2015). Anti-thymocyte globulin/G-CSF treatment preserves beta cell function in patients with established type 1 diabetes. J Clin Invest.

[57] Nepom GT, Ehlers M, Mandrup-Poulsen T (2013). Anti-cytokine therapies in T1D: concepts and strategies. Clin Immunol.

[58] Hundhausen C, Roth A, Whalen E (2016). Enhanced T cell responses to IL-6 in type 1 diabetes are associated with early clinical disease and increased IL-6 receptor expression. Sci Transl Med.

[59] Yu A, Snowhite I, Vendrame F (2015). Selective IL-2 responsiveness of regulatory T cells through multiple intrinsic mechanisms supports the use of low-dose IL-2 therapy in type 1 diabetes. Diabetes.

[60] Koreth J, Matsuoka K, Kim HT (2011). Interleukin-2 and regulatory T cells in graft-versus-host disease. N Engl J Med.

[61] Kennedy-Nasser AA, Ku S, Castillo-Caro P (2014). Ultra low-dose IL-2 for GVHD prophylaxis after allogeneic hematopoietic stem cell transplantation mediates expansion of regulatory T cells without diminishing antiviral and antileukemic activity. Clin Cancer Res.

[62] Saadoun D, Rosenzwajg M, Joly F (2011). Regulatory T-cell responses to low-dose interleukin-2 in HCV-induced vasculitis. N Engl J Med.

[63] Humrich JY, von Spee-Mayer C, Siegert E (2015). Rapid induction of clinical remission by low-dose interleukin-2 in a patient with refractory SLE. Ann Rheum Dis.

[64] Castela E, Le Duff F, Butori C (2014). Effects of low-dose recombinant interleukin 2 to promote T-regulatory cells in alopecia areata. JAMA Dermatol.

[65] Hartemann A, Bensimon G, Payan CA (2013). Low-dose interleukin 2 in patients with type 1 diabetes: a phase 1/2 randomised, double-blind, placebo-controlled trial. Lancet Diabetes Endocrinol.

[66] Long SA, Rieck M, Sanda S (2012). Rapamycin/IL-2 combination therapy in patients with type 1 diabetes augments Tregs yet transiently impairs beta-cell function. Diabetes.

[67] Long SA, Buckner JH, Greenbaum CJ (2013). IL-2 therapy in type 1 diabetes: “Trials” and tribulations. Clin Immunol.

[68] Waldron-Lynch F, Kareclas P, Irons K (2014). Rationale and study design of the adaptive study of IL-2 dose on regulatory T cells in type 1 diabetes (DILT1D): a non-randomised, open label, adaptive dose finding trial. BMJ Open.

[69] Truman LA, Pekalski ML, Kareclas P (2015). Protocol of the adaptive study of IL-2 dose frequency on regulatory T cells in type 1 diabetes (DILfrequency): a mechanistic, non-randomised, repeat dose, open-label, response-adaptive study. BMJ Open.

[70] Marek-Trzonkowska N, Mysliwiec M, Dobyszuk A (2012). Administration of CD4+CD25highCD127- regulatory T cells preserves beta-cell function in type 1 diabetes in children. Diabetes Care.

[71] Marek-Trzonkowska N, Mysliwiec M, Dobyszuk A (2014). Therapy of type 1 diabetes with CD4(+)CD25(high)CD127-regulatory T cells prolongs survival of pancreatic islets - results of one year follow-up. Clin Immunol.

[72] Bluestone JA, Buckner JH, Fitch M (2015). Type 1 diabetes immunotherapy using polyclonal regulatory T cells. Sci Transl Med.

[73] Tang Q, Henriksen KJ, Bi M (2004). In vitro-expanded antigen-specific regulatory T cells suppress autoimmune diabetes. J Exp Med.

[74] Masteller EL, Warner MR, Tang Q, Tarbell KV, McDevitt H, Bluestone JA (2005). Expansion of functional endogenous antigen-specific CD4+CD25+ regulatory T cells from nonobese diabetic mice. J Immunol.

[75] Tarbell KV, Yamazaki S, Olson K, Toy P, Steinman RM (2004). CD25+ CD4+ T cells, expanded with dendritic cells presenting a single autoantigenic peptide, suppress autoimmune diabetes. J Exp Med.

[76] Putnam AL, Safinia N, Medvec A (2013). Clinical grade manufacturing of human alloantigen-reactive regulatory T cells for use in transplantation. Am J Transplant.

[77] Walker MR, Kasprowicz DJ, Gersuk VH (2003). Induction of FoxP3 and acquisition of T regulatory activity by stimulated human CD4+CD25- T cells. J Clin Invest.

[78] Fantini MC, Becker C, Monteleone G, Pallone F, Galle PR, Neurath MF (2004). Cutting edge: TGF-beta induces a regulatory phenotype in CD4+CD25- T cells through Foxp3 induction and down-regulation of Smad7. J Immunol.

[79] Allan SE, Alstad AN, Merindol N (2008). Generation of potent and stable human CD4+ T regulatory cells by activation-independent expression of FOXP3. Mol Ther: J Am Soc Gene Ther.

[80] Allan SE, Song-Zhao GX, Abraham T, McMurchy AN, Levings MK (2008). Inducible reprogramming of human T cells into Treg cells by a conditionally active form of FOXP3. Eur J Immunol.

[81] Brusko TM, Koya RC, Zhu S (2010). Human antigen-specific regulatory T cells generated by T cell receptor gene transfer. PLoS One.

[82] Hoffmann P, Eder R, Boeld TJ (2006). Only the CD45RA+ subpopulation of CD4+CD25high T cells gives rise to homogeneous regulatory T-cell lines upon in vitro expansion. Blood.

[83] Scotta C, Esposito M, Fazekasova H (2013). Differential effects of rapamycin and retinoic acid on expansion, stability and suppressive qualities of human CD4(+)CD25(+)FOXP3(+) T regulatory cell subpopulations. Haematologica.

[84] Tian J, Clare-Salzler M, Herschenfeld A (1996). Modulating autoimmune responses to GAD inhibits disease progression and prolongs islet graft survival in diabetes-prone mice. Nat Med.

[85] Tisch R, Liblau RS, Yang XD, Liblau P, McDevitt HO (1998). Induction of GAD65-specific regulatory T-cells inhibits ongoing autoimmune diabetes in nonobese diabetic mice. Diabetes.

[86] Tisch R, Wang B, Weaver DJ (2001). Antigen-specific mediated suppression of beta cell autoimmunity by plasmid DNA vaccination. J Immunol.

[87] Gibson VB, Nikolic T, Pearce VQ, Demengeot J, Roep BO, Peakman M (2015). Proinsulin multi-peptide immunotherapy induces antigen-specific regulatory T cells and limits autoimmunity in a humanized model. Clin Exp Immunol.

[88] Ludvigsson J, Faresjo M, Hjorth M (2008). GAD treatment and insulin secretion in recent-onset type 1 diabetes. N Engl J Med.

[89] Ludvigsson J, Krisky D, Casas R (2012). GAD65 antigen therapy in recently diagnosed type 1 diabetes mellitus. N Engl J Med.

[90] Pihl M, Akerman L, Axelsson S (2013). Regulatory T cell phenotype and function 4 years after GAD-alum treatment in children with type 1 diabetes. Clin Exp Immunol.

[91] Thrower SL, James L, Hall W et al (2009) Proinsulin peptide immunotherapy in type 1 diabetes: report of a first-in-man Phase I safety study. Clin Exp Immunol 155:156–165 10.1111/j.1365-2249.2008.03814.x PMC2675245 19040615

[92] Unger WW, Laban S, Kleijwegt FS, van der Slik AR, Roep BO (2009) Induction of Treg by monocyte-derived DC modulated by vitamin D3 or dexamethasone: differential role for PD-L1. Eur J Immunol 39:3147–3159 10.1002/eji.200839103 19688742

[93] Herold KC, Gitelman SE, Willi SM (2013). Teplizumab treatment may improve C-peptide responses in participants with type 1 diabetes after the new-onset period: a randomised controlled trial. Diabetologia.

[94] Beringer DX, Kleijwegt FS, Wiede F (2015). T cell receptor reversed polarity recognition of a self-antigen major histocompatibility complex. Nat Immunol.

[95] Kleijwegt FS, Laban S, Duinkerken G (2011). Transfer of regulatory properties from tolerogenic to proinflammatory dendritic cells via induced autoreactive regulatory T cells. J Immunol.

[96] Cerosaletti K, Schneider A, Schwedhelm K (2013). Multiple autoimmune-associated variants confer decreased IL-2R signaling in CD4+ CD25(hi) T cells of type 1 diabetic and multiple sclerosis patients. PLoS One.

[97] Glisic S, Klinker M, Waukau J (2009). Genetic association of HLA DQB1 with CD4+CD25+(high) T-cell apoptosis in type 1 diabetes. Genes Immun.

[98] Birebent B, Lorho R, Lechartier H et al (2004) Suppressive properties of human CD4^+^CD25^+^ regulatory T cells are dependent on CTLA-4 expression. Eur J Immunol 34:3485–3496 10.1002/eji.200324632 15484187

[99] Grossman WJ, Verbsky JW, Barchet W, Colonna M, Atkinson JP, Ley TJ (2004) Human T regulatory cells can use the perforin pathway to cause autologous target cell death. Immunity 21: 589–601 10.1016/j.immuni.2004.09.002 15485635

[100] Nakamura K, Kitani A, Fuss I et al (2004) TGF-β 1 plays an important role in the mechanism of CD4^+^CD25^+^ regulatory T cell activity in both humans and mice. J Immunol 172:834–842 10.4049/jimmunol.172.2.834 14707053

[101] Collison LW, Vignali DA (2011) In vitro Treg suppression assays. Methods Mol Biol 707:21–37 10.1007/978-1-61737-979-6_2 PMC3043080 21287326

[102] Gagliani N, Magnani CF, Huber S et al (2013) Coexpression of CD49b and LAG-3 identifies human and mouse T regulatory type 1 cells. Nat Med 19:739–746 10.1038/nm.3179 23624599

[103] Bacchetta R, Bigler M, Touraine JL et al (1994) High levels of interleukin 10 production in vivo are associated with tolerance in SCID patients transplanted with HLA mismatched hematopoietic stem cells. J Exp Med 179:493–502 10.1084/jem.179.2.493 PMC2191349 7905018

[104] Groux H, O'Garra A, Bigler M et al (1997) A CD4^+^ T-cell subset inhibits antigenspecific T-cell responses and prevents colitis. Nature 389:737–742 10.1038/39614 9338786

[105] Barrat FJ, Cua DJ, Boonstra A et al (2002) In vitro generation of interleukin 10- producing regulatory CD4^+^ T cells is induced by immunosuppressive drugs and inhibited by T helper type 1 (Th1)- and Th2-inducing cytokines. J Exp Med 195:603–616 10.1084/jem.20011629 PMC2193760 11877483

[106] Brun V, Bastian H, Neveu V, Foussat A (2009) Clinical grade production of IL-10 producing regulatory Tr1 lymphocytes for cell therapy of chronic inflammatory diseases. Int Immunopharmacol 9: 609–613 10.1016/j.intimp.2009.01.032 19539556

[107] Brun V, Neveu V, Pers YM et al (2011) Isolation of functional autologous collagen-II specific IL-10 producing Tr1 cell clones from rheumatoid arthritis blood. Int Immunopharmacol 11: 1074–1078 10.1016/j.intimp.2011.03.001 21406270

