# Supplementary material for: Regulatory T cell dysfunction in type 1 diabetes: what’s broken and how can we fix it?
Source: Diabetologia. 2017 Aug 2;60(10):1839–50. doi: 10.1007/s00125-017-4377-1 (PMC6448885; doi:10.1007/s00125-017-4377-1)
Supplement: Supplementary file 1 — (PPTX 300 kb) [file 125_2017_4377_MOESM1_ESM.pptx]

## Slide 1
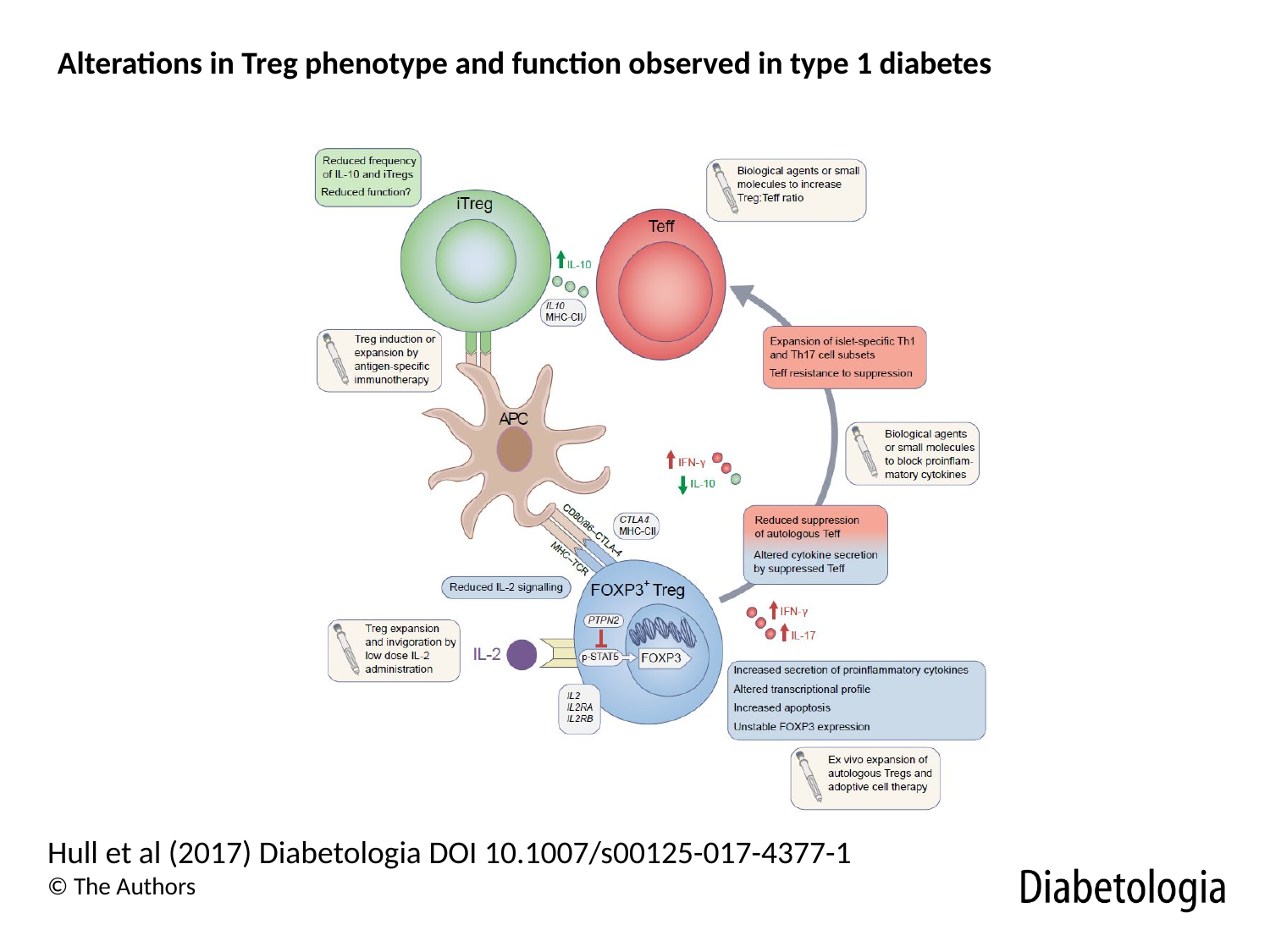

Alterations in Treg phenotype and function observed in type 1 diabetes
Hull et al (2017) Diabetologia DOI 10.1007/s00125-017-4377-1
© The Authors
